# The WHO Prison Health Framework: a framework for assessment of prison health system performance

**DOI:** 10.1093/eurpub/ckac020

**Published:** 2022-04-04

**Authors:** Filipa Alves da Costa, Marieke Verschuuren, Yanina Andersen, Sunita Stürup-Toft, Daniel Lopez-Acuña, Carina Ferreira-Borges

**Affiliations:** WHO European Office for the Prevention and Control of Noncommunicable Diseases, WHO Regional Office for Europe, Moscow, Russian Federation; WHO European Office for the Prevention and Control of Noncommunicable Diseases, WHO Regional Office for Europe, Moscow, Russian Federation; WHO European Office for the Prevention and Control of Noncommunicable Diseases, WHO Regional Office for Europe, Moscow, Russian Federation; Department of Global Operations, UK Health Security Agency, London, UK; Department of International Health, Andalusian School of Public Health, Granada, Spain; WHO European Office for the Prevention and Control of Noncommunicable Diseases, WHO Regional Office for Europe, Moscow, Russian Federation

## Abstract

**Background:**

The Health in Prisons European Database (HIPED) aims to periodically collect data on prison health systems, services and health outcomes to inform equivalence of care for people living in prison. Recognized as the United Nations hub for health data in prisons, HIPED lacked an established framework to define its domains and indicators to measure progress. Therefore, the objectives of developing this framework were to inform surveillance systems at prison, local, regional, national and international level and to use it to guide improvement of prison health systems and cross-country comparison.

**Methods:**

The framework was conceptualized through identification of policy priorities and existing frameworks, notably the WHO Health System Framework. A consultation with a range of WHO stakeholders was conducted evaluating the components of existing frameworks and their relevance to the prison health context, as well as identifying areas needing further emphasis. The final stage identified the structure of the framework.

**Results:**

The framework consists of three main building blocks. The first captures the system-level aspects of prison health care (or inputs) whilst the second captures delivery aspects of prison health care (or outputs). These building blocks are in turn modified by two influencing factors. Ultimately, all these elements impact on the third building block, health outcomes. In addition, two cross-cutting principles associated with all these building blocks and influencing factors are included.

**Conclusions:**

A new framework for assessing prison health system performance is now available, crucial to support informed decision-making for policy design and implementation for prisons and other places of detention.

## Introduction

Universal health coverage (UHC) is central to better health and well-being for all and delivers gains across the 2030 Agenda for Sustainable Development, embodying the pledge to leave no one behind.^1^ The ‘triple billion target’ outlined in the WHO Thirteenth General Programme of Work, adopted at the 2019 World Health Assembly, will only be met by including marginalized and vulnerable groups, such as people living in prison.^2^

People in prison come from the community and, in most cases, return to the community, therefore a period of incarceration is a unique moment to address health inequalities and deliver health interventions. Globally, it is estimated that 11 million people live in prison, with more than 30 million moving between their communities and prisons annually.^3^ The health profile of people in prison is complex, with co-occurring physical and mental health conditions. The link between socio-economic status and unhealthy behaviours is well documented. Data suggests the risk of incarceration is highly dependent on education and ethnicity, and prison populations comprise disproportionately more people with a lower socio-economic status.^4^ There is data to suggest that people with low socio-economic status are more likely to receive heavier sentences.^5^ Evidence shows that substance use (illegal drugs and alcohol) and related disorders are highly prevalent among people in prison.^6^ Also, a strikingly high proportion of the homeless population have substance use disorders, often leading to incarceration. Recently released detainees often have few opportunities to find employment or accommodation, leaving them trapped in poverty and fuelling recidivism. The process of release back into the community provides an opportunity to deliver interventions that could reduce health inequities and the avoidable costs resulting from poor health status and recidivism.

To realize the ambitions of UHC and reduce health inequalities, we need the capacity to measure health system performance to inform evidence-based policy decisions. Various frameworks have been proposed by WHO^7–9^ and other entities^10^^,^^11^ to monitor and measure health-care delivery in a standardized way that allows comparisons to be made between Member States.

Frameworks for mapping health systems are commonly made up of domains representing the main functions and components of such systems, describing inputs and processes, outputs and impacts. The focus of these frameworks can vary from UHC, performance measures or quality indicators. Importantly, these frameworks fail to capture the complexity of health-care delivery in detention settings.

To address this disparity for prison health, the WHO Regional Office for Europe, in collaboration with the UK Collaborating Centre for the WHO Health in Prisons Programme (HIPP) and members of the HIPP Steering Group, developed the Health in Prisons European Database (HIPED). This database gathers information through periodic surveys that collect data on key indicators organized in eight major domains: prison population statistics, prison health-care systems, prison environment, risk factors for ill health, disease screening on admission, prevention of infection, treatment and mortality. Data collected enables understanding of the level of provision of different interventions and the quality of health care, providing Member States with vital information to guide prison health system improvements.^12^ Based on the experience of the first round of HIPED data collection in 2016, the process was further refined through the development of a prison health system performance framework. The framework has been developed to capture the specificities of prison health systems to ensure more effective and systematic data collection for HIPED in 2021.

## Methods

In developing the WHO Prison Health Framework five priorities were identified: strengthen prison information systems, monitor health service provision, track performance, obtain valid and reliable measures of the health status of people living in prison, and conduct intersectoral work (text box 1).

### Framework conceptualization

The framework was developed by MV in close collaboration with FAC and SST, with conceptual inputs from CFB and DLA. First, the literature was reviewed to identify existing frameworks to map health system functions and measure health system performance. This resulted in several existing WHO frameworks being used as a starting point for the conceptualization process—most notably, the well-established Health System Framework.^9^ The structure of this framework encompasses:^1^ Service delivery;^2^ Health workforce;^3^ Information;^4^ Medical products, vaccines and technologies;^5^ Sustainable financing and social protection; and^6^ Leadership and governance. It became apparent that these domains did not address the specificities of prison health systems, however some of its domains were retained using different terminology and/or placed differently to better represent prison health systems.

A consultation with a broad range of external and internal WHO stakeholders was conducted on the components of all frameworks evaluated, the extent of their relevance to the prison health context, and the areas that needed to be emphasized in order to capture the specificities of the prison health system context. This ensured the framework accurately reflects core health system elements while taking into account factors unique to the prison context. Next, the framework’s domains were populated with indicators, which formed the basis for the 2021 round of HIPED data collection (survey available as Supplementary appendix S1). An overview of all the indicators is available in the full report published at WHO’s website^13^ and table 1 provides a summary of the main indicators.

**Table 1:** Example of indicators for each domain and building block of the WHO Prison Health Framework

Building block (BB)	Domain	Indicators
BB: health system	Organization	Prison health-care governance: agency/ministry responsible
		Inspection of prison hygiene, nutrition and living conditions
	Financing	Coverage of prison health care by national health insurance programme (including national health service, if applicable)
		Coverage of community health care by national health insurance programme (including national health service, if applicable)
	Vision and strategy	Implementation of prison health strategy
		Evidence of use of prison health data for planning purposes
	Health information	Existence of a system for recording deaths in custody and parameters included (e.g. cause of death)
		Integration of prison information in the national health information system and systems in place for transferring information to national system
BB: health service delivery	Preventive services: disease prevention	Availability of screening for selected cancers
		Provision of immunization services against vaccine-preventable diseases in line with national vaccination plan
	Preventive services: health protection	Needle/syringe availability
		Personal protective equipment (e.g. hand sanitizer, face masks)
	Preventive services: health promotion	Smoke-free policies implemented
		Policies in place for promotion of physical activity
	Rehabilitation	Availability of user-driven treatment and recovery plans
		Availability of educational and employment training programmes
	Medical care: provision of primary care	Provision of primary care for communicable diseases, including access to and completion of treatment
		Provision of primary care for mental health disorders, including access to treatment
	Medical care: arrangements for secondary and tertiary care	Diversion to specialized treatment for mental health disorders
		Diversion to specialized cancer treatment
	Medical care: continuity of care	Medication reconciliation at admission
		Protocols for continuity of care, including establishment of shared care plans
	Health system performance: availability	Workforce
	Health system performance: accessibility	Out-of-pocket payments for services or health-related products
	Health system performance: acceptability	Consent for health tests, assessments and interventions
	Health system performance: quality of care	Assessments of the availability of essential medicines
BB: health outcomes	Health and well-being	Self-reported health status and well-being
		Access to mental health counsellors
	Morbidity	Mental disorder cases, including psychotic disorder cases, and suicide attempts
		NCD cases, including hypertension, CVD, diabetes and cancer
	Mortality	Number of deaths in prison by any cause (all-cause mortality)
		Number of COVID-19-related deaths (specific indicator developed for 2020/2021)
Influencing factors	Prison environment	Overcrowding
		Availability of basic and improved sanitation
	Health behaviours	Alcohol use
		Physical activity (exercise routines)
Cross-cutting principles (CCP)	Adherence to international standards for human rights and good prison health	Workforce accreditation, professional and ethical standards and their equivalence with the outside community Clinical independence Existence of complaints system
	Reducing health inequalities and addressing the needs of special populations	National standards to meet the health needs of vulnerable people (women, children and youth, LGBTIQ, foreign nationals, ethnic minorities, people who use drugs, elderly, people with disabilities) Meeting the needs of women in prison [e.g. pregnancy tests offered and deliveries (births) in prison]

## Results

### Framework structure

**Figure 1:**
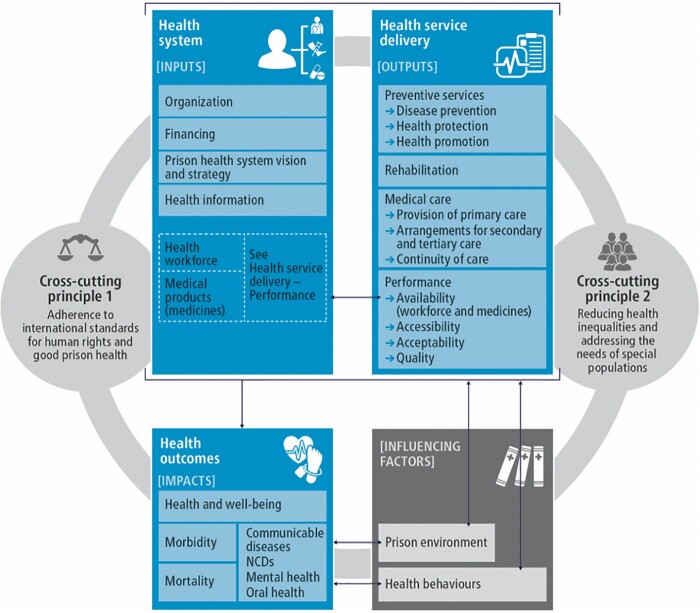
The WHO Prison Health Framework

The first main building block in the WHO Prison Health Framework captures the system-level aspects of prison health care (or inputs); the second block captures provision/delivery aspects of prison health care (or outputs) (figure 1). These building blocks are in turn modified by two influencing factors. Ultimately, all these elements impact on the health outcomes block.

In addition, there are two cross-cutting principles that are specific to the prison context, and influence all major domains mentioned above. These are ‘Adherence to international standards for human rights and good prison health’ and ‘Reducing health inequalities and addressing the needs of special populations’.

To complete the WHO Prison Health Framework, the domains ‘Prison environment’ and ‘Health behaviours’ were included. These two influencing factors modify the way the system’s inputs and outputs translate into health outcomes.

The various elements in the WHO Prison Health Framework were defined based on their specific relevance to prison health systems and further rationale for their selection is explained below.

### Domains of the WHO Prison Health Framework

#### Building block 1. Health system

The first main building block comprises inputs into the prison health system. These include health system organization, financing, and the resources, actors and institutions related to the organization.^7^

##### Organization

Prison health systems vary between and within countries, and there are no ‘universal blueprints’ to determine how to manage and deliver prison health care.^12^ Therefore, information on how the system is organized is required to understand each system’s specific context. Funding arrangements are also diverse, differing on the availability of adequate funding and the source(s) of funding. Leadership and governance ensure that strategic policy frameworks exist and are combined with effective oversight, coalition building, provision of appropriate regulations and incentives, attention to system design and accountability.^8^ In the prison context, different governance arrangements may be in place, with health, justice or interior ministries commonly responsible, impacting on the organization, funding and strategic vision for prison health. A description of the organization of the prison health system and how it compares to the organization of the health system available to the general (non-prison) population is essential to assess equivalence of care and equity in provision.

##### Financing

When a person is deprived of liberty, governments assume responsibility for individuals who are no longer able to seek work and support themselves financially. Therefore, information on financing for prison health is needed, including whether people in prison are covered by existing national health services or insurance schemes available to the general population. Broadly speaking, a good health financing system allocates adequate funds for health, in ways that ensure people can use the services they need and are protected from financial hardship.^8^ Many imprisoned individuals come from marginalized groups of society that may experience barriers in accessing the social care and protection mechanisms that should exist to ensure everyone has the right and opportunity to use health care. In prison, every individual should be granted the financial protection to overcome these potential barriers to access.

##### Vision and strategic approach

To understand the relevance of prison health and the investment that governments make in it, identifying a vision and strategic approach to prison health, embedded in national documents is important. Health strategies play an essential role in defining strategic direction for a health system to ensure the health of its population.^14^ Setting overarching outcomes for the health system is one of the most crucial aspects associated with accountability mechanisms.^15^ Accountability is an important aspect of health system management, requiring authorities to answer questions about their decisions.^16^ This ensures transparency and quality assurance of health-care services, which is especially important in a closed environment.

##### Health information

The Helsinki Conclusions underline the centrality of evidence in improving policy and practice, stating that it is critical to sustain efforts to improve surveillance, to create prison health datasets at national or subnational level, to provide research that can inform decision-making, to conduct systematic evaluations and to document best practices.^2^ A lack of integration of prison and community health information systems has implications for integrated provision and continuity of care during and after release.

#### Building block 2. Health service delivery

##### Preventative services and medical care

Health services may be described as any set of activities whose primary intent is to achieve a state of complete physical, mental and social well-being.^17^ This includes services that are aimed at preventing and treating disease.^18^ Good health services are those that deliver effective, safe, high-quality health interventions, including face-to-face and remotely, to those that need them, when and where needed, with minimum waste of resources.^9^ The main elements of health service provision in prisons are primary care, secondary care and preventive services, such as screening and immunization programmes, and those addressing lifestyle risk factors, including drug use. Prison health services must also have good access to specialized care to ensure that people in prison get the treatment they require and can be transferred to appropriate institutions when needed.^19^

Continuity of care is a crucial element of sustainable health outcomes. Arrangements should be made for continuous access to care at the point of admission, transfer and release, and this should be facilitated by prison management. Continuity of care between prisons and the outside community requires establishment of close organizational relationships between health and social services in prisons and in the community.^2^^,^^19^

##### Rehabilitation

Considering health in its broadest concept,^17^ rehabilitation of people deprived of liberty is crucial to achieving mental and social well-being. Rehabilitation is related to health resilience and can be an important part of the resettlement process on release. Reducing reoffending through rehabilitation programmes is therefore a central goal of the correctional system. These include a broad array of programmes, such as mental health, substance use, educational services^20^ and employment skills development. Rehabilitation is an element of the whole-prison approach to create the best conditions for good health and effective health care.^19^ Although rehabilitation may not, in most health systems, be considered a health service *per se*, certain aspects overlap, and will ultimately influence health outcomes. The issue of rehabilitation in prison health reflects an understanding of the wider determinants of health,^21^ which include education, training, employment opportunities and social relationships.

##### Health system performance

The final domain within the Health Service Delivery building block comprises aspects of health system performance. While the other domains in this building block tell us something about ‘what’ health services are being delivered, this domain tells us something about ‘how’ they are being delivered, and whether this meets existing standards and/or expectations. Measuring health system performance is a key component of accountability mechanisms in health systems.^15^ The four main aspects of prison health system performance are availability, accessibility, acceptability and quality. Availability involves having sufficient facilities, services and goods and having adequate personnel resources to deliver. The ability of systems to respond is highly dependent on workforce and its optimization.^9^^,^^22^ As an example, the health workforce in the prison setting should include, not only physicians and nurses, but dentists and specialists in certain areas of medicine (such as psychiatry), and other healthcare professionals (such as psychologists), determined by the profile of the prison population and its needs which will include mental health and/or substance use disorders. Certain services may need to be provided by non-governmental organizations, for instance, needle and syringe exchange programmes. Accessibility relates to these facilities, services and goods, including health-related information, being physically and economically accessible without discrimination, especially to vulnerable or marginalized populations. Acceptability addresses the extent to which the facilities, services and goods respect medical ethics, confidentiality and the principles of benevolence and non-maleficence to the recipient of care. It also considers the extent to which these services are acceptable to the population benefiting from them and is thus embedded in the principles of autonomy and person-centred care. Finally, quality considers the scientific and medical appropriateness of facilities, services and goods in terms of quality standards.^23^ High-quality health services embrace a person-centred approach; therefore, prison health services must also be person-centred to meet the needs of justice-involved individuals.^2^

#### Building block 3. Health outcomes

The third building block translates the investments made in the health system (Block 1) and the delivery of health services (Block 2) into health outcomes, and comprises three domains: health and well-being, morbidity and mortality. Health and well-being are concepts that arise from the WHO definition of health^17^ but their use has been limited in settings where people have reduced agency and may become victims of abuse. The inclusion of a domain capturing these aspects of health is therefore included primarily to motivate the adoption of this developing concept. Morbidity and mortality are important measures to assess the health status of a population. In the prison context, they are also important domains to evaluate the health system’s performance in offering equal opportunities for those incarcerated compared to those in the outside community.

Morbidity indicators may be divided into two major groups—communicable diseases and non-communicable diseases (NCDs) both of which have been shown to have a heavier burden on prison populations compared to the wider community in many countries.^24^ In particular, mental health and substance use disorders in the prison context assume greater predominance than in the community.^6^^,^^25^

Mortality indicators are associated with the impact of the prison environment and of care received during imprisonment. The mortality domain is one that extends beyond the period of incarceration as it reflects the ultimate impact of the incarceration experience and may only be possible to determine following release.

#### Influencing factor 1. Prison environment

The environment in which people in prison live is an important determinant of health. Important aspects of a healthy environment include accommodation that offers enough space, light and fresh air; good hygiene and clean sanitary facilities; clothing and heating suitable for the climate; and adequate nutrition adapted to individual needs. These aspects are captured in this influencing factor and may be operationalized through various indicators, such as overcrowding, which encapsulates the influence of the physical and social environment, both of which impact on health outcomes.^19^

#### Influencing factor 2. Health behaviours

Unhealthy behaviours are common among people in prison coming from disadvantaged backgrounds. Both drugs and alcohol are associated with offences and there is a disproportionate use of them by people in prison compared to the general population.^26^^,^^27^ Smoking prevalence in prisons is more than three times higher than in the general population.^24^^,^^28^ Drug use may continue inside prison with potentially riskier patterns of use due to the prison environment. However, a period in prison may also present an opportunity to adopt a healthier lifestyle when a health-promoting environment exists. This influencing factor of the framework captures the effect of the services that are available, including health promotion activities, and of the prison environment in the adoption of healthy behaviours, which may change over the course of the incarceration period, impacting on the health outcomes domain of the framework.

#### Cross-cutting principle 1. Adherence to international standards for human rights and good prison health

Internationally agreed principles on the treatment of people in prison play an important role in prison health. The Mandela Rules^29^ clearly state that deprivation of liberty is itself the punishment for crime; respect for human dignity and fundamental human rights, including equal standards between prison and community health care and clinical independence of health-care staff, must always be observed during imprisonment. Clinical independence refers to health-care staff having the freedom to exercise their professional judgement in the care and treatment of their patients without undue or inappropriate influence by outside parties or individuals, such as justice administrators. An essential component of high-quality medical care is a patient–caregiver relationship built on trust and professionalism. Assessing the extent to which prison health systems adhere to these principles is therefore an important measure of the functioning and quality of these systems.

#### Cross-cutting principle 2. Reducing health inequalities and addressing the needs of special populations

The second cross-cutting theme deals with inequalities and the needs of special populations, mostly those that may be victims of discrimination. Reducing health inequalities by addressing the health needs of special populations, including women, young, elderly and disabled people, people who use drugs and people who are lesbian, gay, bisexual, transgender, intersex and queer (LGBTIQ), as well as foreign nationals and non-native speakers, should be one of the priority areas for prison health care^19^ and prison health governance. Tailoring interventions to meet the needs of special populations is required of good health service provision.

As an illustration of this cross-cutting principle, women in prison present an especially vulnerable and complex profile, often with experiences of past violence and trauma.^30^ Women can be held in separate prisons, which are often set up for the needs of men and thus not well adapted to their needs,^31^ including access to family and social networks. Female prisons also affect health workforce organization, as women have the right to demand a female physician and/or nurse to examine them.^32^ There are also basic needs and specific services that need to be available to this population, including reproductive health planning and availability of sanitary towels or tampons. Women in prison have marked excess substance dependence, including alcohol and illicit drugs, and are more prone to obesity or overweight when compared to the general population,^33–35^ which suggests that the priorities for intervention in female prisons should include nutrition and exercise, as well as alcohol and drug use/harm minimization interventions.

## Discussion

The WHO Prison framework fills in an important gap in public health. It will enable assessing prison health services and performance at international level and use data obtained to benchmark performance between countries to improve health outcomes for this vulnerable population. The next step is a final validation of the refined indicators to ensure they adequately reflect all major domains of the framework. During 2021, this improved version of HIPEDS was sent to all 53 Member States, via their Ministries of Health, to obtain data that may inform the current status of prison health performance in the WHO European Region. The value of having an instrument, underpinned by a theoretical framework, that allows cross-country comparisons is unvaluable. Results obtained will be used to work in close collaboration with Member States to identify priorities for investment in their prison system, including information systems, so that people deprived of liberty may in the future achieve improved health outcomes. Further improvements planned for subsequent editions include the addition of a glossary, which may facilitate understanding all variables included.

Notwithstanding the value of HIPEDS, it must be recognized that not all areas can be sufficiently detailed considering the overarching ambition of the framework to measure the performance of the prison health system. For instance, the survey currently collects little information on drug-related interventions in prison and this may be seen as a limitation considering the high proportion of drug-related offences resulting in incarceration. However, other organizations specializing in drug use policies, such as the EMCDDA and UNODC, periodically collect in depth data on such indicators. The changing profile of people in prison requires focus on other areas that have traditionally been neglected for this population. For example, the ageing prison population necessitates more detailed indicators related to NCDs. The specific items that are included in HIPEDS may however, be modified in future editions, reflecting population trends and the framework’s ambition to encompass the full remit of prison health services.

In conclusion, improving information systems across the European Region is crucial for the development of evidence-based policies. Various frameworks proposed by WHO and others to monitor and measure health-care delivery in a standardized way enable comparisons of the effectiveness of different policy approaches on health system performance between Member States. However, these frameworks often fail to capture the complexity of health-care delivery in prisons and other places of detention. The WHO Prison Health Framework is expected to support Member States in systematically measuring and documenting the performance of their prison health systems and the health status of their prison populations at country level, thereby helping to ensure that all people in prison achieve the highest standard of health, regardless of race, religion, political belief, or economic and social condition.

## Supplementary data

Supplementary data are available at *EURPUB* online.

## Funding

The publication was made possible by funding from the Ministry of Social Affairs and Health of Finland.

## Disclaimer

C.F.-B. is a staff member of the WHO; F.A.C., M.V., Y.A. and D.L.-A. are WHO consultants. The authors alone are responsible for the views expressed in this publication and these do not necessarily represent the decisions or the stated policy of the World Health Organization.

*Conflicts of interest*: None declared.

Key points

- The investment in health information is a priority for evidence-based policy decisions.
- The new WHO Prison Health Framework was developed to capture the specificities of the prison health system context.
- The implementation of this new framework will enable systematic and standardized assessment of prison health system performance.
- The operationalization of the WHO Prison Health Framework will enable data to inform improvements in health outcomes for people in prison.

## Supplementary Material

ckac020_Supplementary_Data
