# Supplementary material for: The WHO Prison Health Framework: a framework for assessment of prison health system performance
Source: Eur J Public Health. 2022 Apr 4;32(4):565–70. doi: 10.1093/eurpub/ckac020 (PMC9341673; doi:10.1093/eurpub/ckac020)
Supplement: ckac020_Supplementary_Data [file ckac020_supplementary_data.pdf]

National questionnaire for minimum public health dataset for prisons in the WHO European Region

The Health in Prisons European Database (HIPED), is an initiative led by WHO Europe to build on evidence around the health of people in prison and the services provided to them. As part of this initiative, we request Member States to periodically provide data to contribute to HIPED through a survey.

The current survey builds on the WHO Framework for Prison health system's performance assessment. This framework describes eight domains for which a selected list of key indicators has been identified to reflect the prison environment, issues of availability, accessibility, care provision, health behaviours and health outcomes. It is a long survey, but not exhaustive so that all domains may be captured.

Guidance on completing the questionnaire

This survey is addressed at the survey focal point nominated by the Member State's Ministry of Health. However, you may find it useful to involve other Ministries with responsibility shared for healthcare in prisons, several national experts or an expert group in the completion of this questionnaire. If possible, please list all experts consulted in the section provided below as appropriate, so that they can be acknowledged in the final Report to be produced. We specifically ask for the identification of the survey focal point in case any additional contact is requested to request further clarifications.

1. The data requested refers to the most recent year, *i.e.*, from **01.01.2020 to 31.12.2020**. In case it is not possible to provide such updated data, please refer to the previous homologous period (01.01.2019-31.12.2019) and indicate that in the comments.
2. Where a question refers to the prisoners in your country, please provide a response which applies to **all persons in the prisons of your country**, including those held in pre-trial detention (*e.g.* in remand prison/jail) wherever available. Questions which refer to the prisons of your country likewise require a response which applies to **all the prisons in your country**. Note: even though WHO adopts person-centred wording in all external publications (people living in prisons, for simplicity of the survey, throughout the questions we will use the term "prisoner").
3. Wherever your responses refer to a different base (*e.g.,* prisoners excluding those in pre-trial detention), please indicate this clearly with a comment.
4. This survey **does not refer to other prescribed places of detention** (*e.g.* immigration detention centres and police custody or their equivalent).
5. Where questions ask about national practice, if there is substantial regional variation in practice please provide detail about this in a comment.
6. Where answer categories provided do not apply to the situation in your country, please write a comment.
7. Please document each source that contributed to the data provided, including whether it was obtained from a published report and if so, the nature of that publication (*i.e.,* scientific journal or government agency), and if any data was obtained from an unpublished source. Whenever possible, please indicate the links to the source data.

EXPERTS CONSULTED

For the completion of this survey, comprising the sections: A. Penal Statistics; B. Prison Health Systems; C. Health Services; D. Health Outcomes; E. Prison Environment; F. Health Behaviours; G. Adherence to Equivalence and Other International Standards; and H. Reducing Health Inequalities, you are encouraged to contact and consult additional experts. These experts could come from the following areas:

- Person in charge of or involved in prison health in the Ministry of Health/Ministry of Justice/Ministry of Interior of your country, or the most senior government official in charge of prison health conditions;
- The head of a prominent non-governmental organization dedicated to prison health;
- A health professional (*e.g.*, medical doctor, nurse, pharmacist, social worker, psychologist) specialized in prison health-related services;
- A faculty member of a university department;
- A police or other law enforcement officer;
- A person at the Ministry of Finance, tax agency or statistical office.

For countries with regional or sub-national arrangements, alternatively you can also select experts from each of the different regions and eventually then set up meetings to evaluate and decide the comparability of data and the possibility for national aggregation.

Contact for questions or clarifications: azevedof@who.int

CONTACT INFORMATION

Date: _ _ / _ _ / _ _ _ _ (Day/Month/Year)

WHO Region: ____________________________

Country: ________________________________

Questionnaire completed by:

Last name: _______________________________ **First name:** ________________________

Title/Position: ____

Institute/Ministry/etc. ____

Address: ____

Telephone: _______________________________

Fax: _____________________________________

E-mail: ___________________________________

For those whom you did consult, please enter the following information accordingly:

Expert 1 –

Name:____

Position:____

Organization:____

Expert 2 –

Name:____

Position:____

Organization:____

Expert 3 –

Name:____

Position:____

Organization:____

Comments:____

SECTION A: PENAL STATISTICS

A1. What is the official prison capacity in your country?

Note: The official capacity of a prison is defined by the total number of detainees that it can accommodate while respecting the standards set by the relevant authority in the country. When prison buildings are old, prison administrations are not always able to give figures for the floor space allocated to each detainee or group of detainees. However, the official capacity of prisons at the time of construction is usually known.

	Data	No data
**A2. What is the total number of prisoners in your country by 31.12.2020?**		☐
**A2.1 Among those mentioned in A2, how many are:**<br>a) Female		☐
i) Of those in a), how many were pregnant during the last 12 months?		☐
b) Lesbian, gay, bisexual, transgender, intersex and queer people (LGBTIQ)		☐
c) Young people (under 18 years of age)		☐
d) Older people (above 50 years of age)		☐
e) Older people (above 65 years of age)		☐
f) Migrants (i.e. not national citizens in the country of detention)		☐
g) From an ethnic/racial minority		☐
h) People living with disabilities		☐
i) Physical disabilities		☐
ii) Intellectual disabilities		☐
**A3. What is the number of unsentenced/remand prisoners in your country (excluding those in police custody) by 31.12.2020?**		☐
**A4. What is the number of unique individuals entering prison over the most recent 12-month period (01.01.2020–31.12.2020)?**		☐
**A5. What is the mean number of occasions a unique individual entered prison over the last 12-month period?**		☐

**A6. What was the mean length of incarceration per individual over the last 12-month period** **(please indicate your answer in <u>months</u>)?**		☐
**A7. What is the total number of prison establishments in your country?**		☐

	Data	No data	Not legally permitted in the country
**A8. What is the number of individuals serving life sentences?**		☐	☐

SECTION B: PRISON HEALTH SYSTEMS

HEALTH SYSTEM ORGANIZATION

B1. In your country, what level of government is responsible for prison health care? Please choose the answer that best describes your country. If none of the options perfectly describes it, please choose “other” and specify.

a) National government is responsible for prison healthcare
b) National government and sub-national governments both have responsibilities
c) Sub-national governments have responsibility and national government does not have responsibility
d) Other. Please specify:

B2. In your country, what level of government is responsible for the delivery of health care for the general population (i.e., outside of prisons and can include primary and secondary care)? Please choose the answer that best describes your country. If none of the options perfectly describes it, please choose “other” and specify.

a) National government is responsible for healthcare
b) National government and sub-national governments both have responsibilities
c) Sub-national governments have responsibility and national government does not have responsibility
d) Other. Please specify:

B3. In your country, which agency or agencies are responsible for delivering prison healthcare. Please choose the answer that best describes your country. If none of the options perfectly describes it, please choose “other” and specify.

a) Ministry of Health only (or health authorities)
b) Ministry of Justice only
c) Ministry of Interior only
d) Other ministry in isolation. Please state which:
e) Both Ministry of Health and Ministry of Justice/Ministry of Interior
f) Another situation. Please specify:

B4. In your country, which agency or agencies are responsible for the inspection of prison hygiene, nutrition and living conditions? Please choose the answer that best describes your country. If none of the options perfectly describes it, please choose “other” and specify.

a) Ministry of Health only (or health authorities)
b) Ministry of Justice only
c) Ministry of Interior only
d) Other ministry in isolation or independent organization. Please state which:
e) Both Ministry of Health and Ministry of Justice/Ministry of Interior
f) Another situation. Please specify:

HEALTH SYSTEM FINANCING

B5. In your country, which agency or agencies are responsible for financing prison healthcare (i.e., is responsible for holding and managing the budget for these services). Note: Financing refers to responsibility for managing the funding necessary for prison healthcare services. This may be the responsibility of one or more ministries. It may be the same agency which is responsible for the delivery of prison healthcare services, or a separate agency. **Please choose the answer that best describes your country. If none of the options perfectly describes it, please choose "another situation" and specify.**

a) Ministry of Health only
b) Ministry of Justice only
c) Ministry of Interior only
d) Other ministry in isolation. Please state which:
e) Both Ministry of Health and Ministry of Justice/Ministry of Interior
f) Another situation. Please specify:

B6. To what extent is healthcare of people in prison covered by any health insurance systems (includes the public national health service) which apply to the general (non-prison) community? Please choose the answer (s) that best describes your country. If none of the options perfectly describes it, please choose "another situation" and specify.

a) Healthcare for people in prison is fully covered by health insurance (the same as for the general community)
b) Healthcare for people in prison is partly covered by health insurance (the same as for the general community)
c) Healthcare for people in prison is covered by a separate health insurance system (different to what is available in the general community)
d) Healthcare for people in prison is not covered by any health insurance
e) Another situation. Please describe below the situation in your country:

B7. Are prisoners in your country obliged to cover any of the following expenses?

	**Prisoners cover all costs**	**Prisoners cover some costs**	**Prisoners do not cover any costs**
General healthcare services	☐	☐	☐
Prescription medication	☐	☐	☐
Other expenses (please specify):	☐	☐	☐

HEALTH SYSTEM VISION AND STRATEGY

B8. Is there a national/subnational prison health policy/strategy? Please choose the best answer.

a) Yes, there is a national/subnational prison health policy/strategy. Please attach or provide a link to the relevant document(s):
b) Yes, prison health is part of another national/subnational (health) policy/strategy. Please specify and attach or provide a link to the relevant document(s):
c) No, there is no such policy/strategy at present, but it is envisaged for the future. Please specify and attach or provide a link to draft/plan/other evidence:
d) No, there is no such policy/strategy at present and there is currently no intention to develop one in the immediate future

B8.1 If the answer to B8 is 'Yes' (a or b), is there an implementation plan for the policy/strategy?

a) Yes, implementation plan is already adopted. Please attach or provide link to evidence:

b) Yes, implementation plan is under development or there are plans for development. Please attach or provide link to draft/plan/other evidence:
c) No, there is no implementation plan at present and no such plan will be developed

HEALTH SYSTEM PERFORMANCE

Availability

B9. Please indicate the total number of healthcare staff (physicians, nurses, nursing assistants, etc., including external service providers) in prisons on full-time equivalents (FTEs) for a known year.

Year	
Number	

B9.1 Among those in B9, please indicate the total number of:

	Total Number	No data
Physicians (including external service providers) based FTEs		☐
Nurses (including external service providers) based on FTEs		☐
Psychiatrists (including external service providers) based on FTEs		☐
Dentists (including external service providers) based on FTEs		☐

Acceptability

B10. In case screening tests and/or health assessments are being offered to prisoners, is informed consent being obtained and documented (could be for all health assessments/interventions and not necessarily per assessment/intervention)?

a) Yes, these are obtained
b) Yes, these are obtained and documented
c) No

B11. For each of the following programmes for vaccine-preventable diseases, please indicate the proportion of prison establishments in your country where these are available to be administered to eligible prisoners?

	All prisons	Most prisons	A minority of prisons	No prisons
DTP (diphtheria, tetanus, pertussis)	☐	☐	☐	☐
Human Papilloma virus	☐	☐	☐	☐
Hepatitis A	☐	☐	☐	☐
Hepatitis B	☐	☐	☐	☐
Seasonal flu	☐	☐	☐	☐
MMR (measles, mumps and rubeola)	☐	☐	☐	☐
Meningococcal vaccination	☐	☐	☐	☐
Pneumococcal vaccination	☐	☐	☐	☐
COVID-19	☐	☐	☐	☐

B12. Please indicate the proportion of prison establishments where prisoners have access to HIV prophylaxis?

	All prisons	Most prisons	A minority of prisons	No prisons
Post Exposure	☐	☐	☐	☐
Pre-exposure	☐	☐	☐	☐

Quality of Care

B13. Are regular assessments performed in prisons on the availability of essential medicines?

a) Yes
b) No

B14. Is there a standardized process for reporting medication errors in prisons (Errors or mistakes committed by health professionals which result in harm to the patient, source: https://meshb.nlm.nih.gov/record/ui?ui=D019300)**?**

a) Yes. Please specify:
b) No

B15. Is there a standardized process for reporting adverse drug events in prisons (Disorders that result from the intended use of pharmaceuticals, source: https://meshb.nlm.nih.gov/record/ui?ui=D064420)**?**

a) Yes. Please specify:
b) No

B16. Is there a standardized protocol for identifying and helping people with suicide/self-harm risk in prisons?

a) Yes for suicide
b) Yes for self-harm and suicide
c) No

B17. Is there a mechanism in place for ensuring patient involvement in healthcare planning and reform?

a) Yes. Please specify:
b) No

HEALTH INFORMATION

B18. Is there a registration system for keeping track of deaths in prisons?

a) Yes
b) No

B18.1 If the answer to B18 is 'Yes', does this also include the causes of the deaths that occurred in prison?

a) Yes
b) No

B18.2 Please explain how data on deaths and causes of death are being transferred from the prison registration system to the national Civil Registration and Vital Statistics (CRVS) registration (open question).

B18.3 Are completeness and quality of the data on deaths that are being sent to the national CRVS registration regularly assessed?

a) Yes. Please provide the most recent figure: (%)
b) No

B18.4 Do the physicians that work in the prison health systems receive training for filling in the death certificates?

a) Yes
b) No

B19. Do prisons inform public health authorities about diseases amongst prisoners?

a) Yes
b) No

B19.1 If the answer to B19 is 'Yes', is individual imprisonment status captured in the disease registries or surveillance data (e.g. place of infection, place of diagnosis, risk factor)?

a) Yes, for infectious diseases only (IDs)
b) Yes, for Non communicable Diseases (NCDs) only
c) Yes, both for IDs and for NCDs
d) No

B19.2 If the answer to B19 is 'Yes', please provide the following:

Provide link to NCDs datasets or annual reports	
Provide link to IDs datasets or annual reports	
Indicate completeness of reporting for NCDs (%)	
Indicate completeness of reporting for IDs (%)	

B20. Do you keep clinical health records of people in prison?

a) Yes, we keep paper-based clinical health-records
b) Yes, we keep electronic clinical health records
c) Yes, in some prisons we keep paper-based clinical health-records and in others we keep electronic clinical health records
d) No

B20.1 If the answer to B20 is 'Yes', does the clinical health record system include sections for recording information on (please mark all that apply):

- Screening tests performed
- Screening tests results
- Vaccination (e.g. vaccination history, vaccines administered during incarceration)
- Health behaviours (e.g., tobacco use, alcohol use, drug use)
- Diagnoses established
- Visits to external care providers (e.g., hospital admissions or specialized care appointments)
- Treatment and medications

B20.2 If the answer to B20 is 'Yes', is the clinical health record system used in prisons compatible with the health record system used for the general population in the country?

a) Yes, the same system is being used which is interoperable and allows for individual health data exchange across the community-prison interface
b) Yes, different systems are being used, but these are interoperable and allow for individual health data exchange across the community-prison interface
c) Yes, the same system is used or is interoperable and allows for individual health data exchange across the community-prison interface but only for certain conditions, interventions or population subgroups (e.g., HIV, immunization). Please specify:
d) No

B21. Does your country have the capacity to provide timely (i.e., equivalent to general community standards) **surveillance data of COVID-19 cases identified in prisons (prisoners and custodial staff)?**

a) Yes, we can provide exhaustive data in a timely manner
b) Yes, we can provide exhaustive data but not in a timely manner
c) Yes, we can provide data but not for all variables (e.g., age disaggregation, prisoners, custodial staff)
d) No, we cannot provide data. Please explain why below:

B22. Please indicate if prisons in your country undertake contact tracing in relation to COVID-19 cases?

a) Contact tracing is undertaken in all prisons
b) Contact tracing is undertaken in most prisons
c) Contact tracing is undertaken in a minority of prisons
d) Contact tracing is not undertaken in any prison

B23. If COVID-19 vaccination is implemented in at least one prison in your country, is the immunization information system in prison interoperable with immunization information system in the community?

a) Yes
b) Yes, for some prisons
c) No
d) Not applicable

SECTION C: HEALTH SERVICES

PREVENTIVE SERVICES

Disease Prevention

C1. Is there an initial urgent health needs assessment undertaken in the first 24h after reception?

a) Yes
b) No

C2. Is a more detailed review of health needs subsequently conducted (e.g., within 7 days of admission)?

a) Yes
b) No

C2.1 If the answer to C1 or C2 is ‘Yes’, can you indicate how many unique individuals have received a health examination following admission to prison in the past 12-month period? (Provide number)

C2.2. If the answer to C1 or C2 is ‘Yes’, who conducts these assessments?

a) Nurse only
b) Physician only
c) Nurse predominantly, but with referral to physician available
d) Another healthcare worker. Please indicate who:
e) Member of custodial staff only
f) Other possibility. Please explain:

C2.3 If the answer to C1 or C2 is ‘Yes’, which of the following is part of the assessment? Please mark for each possible assessment, the response option that best describes your country.

	**All prisons**	**Most prisons**	**A minority of prisons**	**No prisons**
Alcohol use	☐	☐	☐	☐
Drug use	☐	☐	☐	☐
Injection drug use	☐	☐	☐	☐
Smoking status	☐	☐	☐	☐
Blood pressure measurement	☐	☐	☐	☐
Body mass index calculation	☐	☐	☐	☐
Mental health problems (e.g., psychosis)	☐	☐	☐	☐
Respiratory conditions (e.g., COPD)	☐	☐	☐	☐
Oral health problems (e.g., tooth decay)	☐	☐	☐	☐

Chronic conditions requiring treatment (e.g. HIV, CVDs)	☐	☐	☐	☐
COVID-status and/or COVID immunization status	☐	☐	☐	☐

C3. Are history of TB and current signs and symptoms assessed on or close to reception for all people in prison?

a) No
b) Yes, a clinical evaluation of signs and symptoms is made, including evaluation of previous history
c) Yes, and a diagnostic test is offered in addition to the clinical evaluation
d) Yes, clinical assessment and diagnostic tests are made and when the test is positive, additional assessment for MDR-TB is ensured

C4. Please mark for the following infectious diseases, if screening is being offered on or close to reception to all prisoners? For each disease, please choose the situation that best describes your country.

	**Yes, on an opt out basis**	**Yes, on an opt in basis**	**Yes, risk-based screening**	**No**
HIV	☐	☐	☐	☐
HCV	☐	☐	☐	☐
HBV	☐	☐	☐	☐
STI	☐	☐	☐	☐

C5. Does your country have any of the following types of cancer screening offered to prisoners?

	**Yes**	**No**
Cervical	☐	☐
Colon	☐	☐
Breast	☐	☐

C6. Do these cancer screenings apply the same eligibility criteria (e.g., age cut-off) as those conducted in the general population (community model) (please choose all that apply)

a) Yes, the same criteria apply in cervical cancer
b) Yes, the same criteria apply in colon cancer
c) Yes, the same criteria apply in breast cancer

C7. Are there any specific restrictions or differences on screening practices for prison and in the community (please choose all that apply).

a) Yes, the methods used are different (e.g., FOBT or FIT vs colonoscopy for colorectal cancer; Pap test vs cytology for cervical cancer)
b) Yes, the frequency used is different (e.g., annual vs biannual; this may happen as a result of availability of mobile units properly equipped)

c) Yes, another situation. Please describe below:
d) No

Health Protection

C8. For each of the following products, please indicate if they are offered free of charge considering the response options given.

	All prisons	Most prisons	A minority of prisons	No prisons
Soap	☐	☐	☐	☐
Condoms	☐	☐	☐	☐
Lubricants	☐	☐	☐	☐
Needles and syringes	☐	☐	☐	☐
Disinfectants (“bleach” to use in needles or piercing/tattooing material)	☐	☐	☐	☐
Dental dams	☐	☐	☐	☐
Tampons/sanitary towels	☐	☐	☐	☐

C9. Since the emergence of COVID-19, did prisoners have access to:

	Yes	No
a) Hand sanitizer/soap and water	☐	☐
b) Face masks	☐	☐

C10. Did the prisons in your country create space for adequate quarantine of contacts and isolation of COVID-19 cases (e.g., single-cell accommodation or multiple occupation by cohorting?
a) In all prisons
b) In most prisons
c) In a minority of prisons
d) In no prisons

C10.1 If the answer to C10 is a, b or c, did this space consider the CPT rules (6m² of living space for a single-occupancy cell – excluding toilet space – and adding 4m² per additional inmate)?
a) In all prisons
b) In most prisons

c) In a minority of prisons
d) In no prisons

Health Promotion

C11. Are there health promotion materials like brochures and leaflets available on safe tattooing practices?

a) Yes
b) No

C12. Are there any policies or procedures in place to promote physical activity in prison?

a) Yes. Please provide link/describe below:
b) No

C13. Do prisons in your country have therapeutic spaces available for people with drug problems?

a) In all prisons
b) In most prisons
c) In a minority of prisons
d) in no prisons

C14. Is there any smoke free policy implemented in your country applicable to prisons?

a) Yes, nationwide
b) Yes, in specific regions of the country
c) No

PROVISION OF PRIMARY CARE

C15. Are there any preparedness contingency plans for managing the impact of an infectious disease outbreak in prisons?

a) In all prisons
b) In most prisons
c) In a minority of prisons
d) In no prisons

C15.1 If the answer to C15 is a, b or c, in case a pandemic response plan has been developed and is published, please indicate the link.

C15.2 If the answer to C15 is a, b or c, in case a policy response plan has been developed for COVID-19 and is published, please indicate the link.

C16. Do suspected cases of an infectious disease have access to laboratory tests?

a) Yes, everyone in prison has access to laboratory tests when these are necessary
b) Yes, but there are limited resources, so only the priority/vulnerable groups have access
c) No

ARRANGEMENTS FOR SECONDARY AND TERTIARY CARE

C17. Are there any arrangements/protocols established to ensure access for people in prison to specialized treatment of mental health disorders?

a) In all prisons
b) In most prisons
c) In a minority of prisons
d) In no prisons

C18. Are there any arrangements/protocols established for transferring people in prison to specialized institutions to treat cancer?

a) In all prisons
b) In most prisons
c) In a minority of prisons
d) In no prisons

CONTINUITY OF CARE

C19. Is there any support service to register people released from prison with a GP/community health services?

a) Yes
b) No

C19.1 If the answer to C19 is 'Yes', does this service include any of the following:

1) Scheduling medical appointment upon release
2) Development of a Care Plan to be shared with external providers

C20. Is there a procedure in place to ensure medication is reconciled (procedure in place for transferring a list of prescribed medication used by new entrants to prisons in the community to the prison healthcare service) **at admission (first 24h)?**

a) Yes
b) No

C21. When people are released from prison, are they provided with any medication?

a) Yes, for all conditions.
b) Yes, for some conditions.
c) No

C21.1 If the answer to C21 is b, medication for, choose what applies:

- Drug Use Disorders
- HIV
- TB
- HCV
- Other disease, please specify which:

C22. When people are released from prison, are they tested for COVID-19 prior to release?

a) Yes
b) No

REHABILITATION

Education and Training

C23. Do people in prison have access to education and training programmes?

a) In all prisons
b) In most prisons
c) In a minority of prisons
d) In no prisons

Employment Opportunities

C24. Do people in prison have access to employment opportunities while in prison?

a) In all prisons
b) In most prisons
c) In a minority of prisons
d) In no prisons

Social Relationships

C25. Please indicate the conditions under which people are allowed to continue their family relationships.

	No	Yes, with time restrictions	Yes, free of charge
By telephone	☐	☐	☐
By web communication	☐	☐	☐
By physical visits on the premises	☐	☐	
By physical visits outside the detention facility	☐	☐	

C26. Are people placed in prisons considering the location of their home to facilitate maintaining family relationships?

a) Yes, always
b) Yes, as much as possible
c) No, allocation is made according to other factors

SECTION D: HEALTH OUTCOMES

HEALTH & WELL BEING

D1. Are assessments of perceived well-being (or life satisfaction) of people in prison conducted?

a) Yes, regularly (for example once every year or once every two years)
b) Yes, on an ad hoc basis
c) No, it has never been done

D2. Do all people in prison have access to mental health counsellors (including peer support and external providers – not specifically for mental health disorders but including support for maintenance of well-being when needed)?

a) In all prisons
b) In most prisons
c) In a minority of prisons
d) In no prisons

MORTALITY

	Data	No data
**D3. Please provide the number of unique individuals who died over past 12 months (any cause)**		☐
**D3.1 Among those in D3, how many died as a result of:**<br>a) Suicide		☐
b) Drug overdose		☐
c) COVID-19		☐
**Please indicate three additional top causes of death:**<br>d) Cause 1:		☐
e) Cause 2:		☐
f) Cause 3:		☐

MORBIDITY

D4. Please fill in the following table, which asks for data concerning medical care provision (3.1), diagnoses established (before and during incarceration – 4.2) and treatment provided (including pharmacological and non-pharmacological). For easier organization, indicators are organized by disease condition. We ask you to indicate in the first column the most recent data (year 2020) and only in case this is unavailable, use the following column and add the reference year indicating the year in the last column. For all data we request you to indicate numbers disaggregated by sex and only for a few conditions (HIV, HCV, HBV and STIs), specify among females the number that were pregnant in the period considered. Please indicate the reference year in the last column, for data provided in the first two columns, only if it does not refer to 2020.

	**2020**				**OTHER REFERENCE YEAR**
**Tuberculosis TB**	**Both sexes**	**M**	**F**		
Number of unique individuals with active TB diagnosis (ICD code A15–19)					
Number of unique individuals receiving TB treatment over the last 12-month period					
Number of unique individuals completing TB treatment over the last 12-month period					
**Multidrug-resistant Tuberculosis (MDRTB)**	**Both sexes**	**M**	**F**		
Number of unique individuals with active MDR-TB diagnosis (ICD code Z16.342)					
Number of unique individuals receiving MDR-TB treatment over the last 12-month period					
Number of unique individuals completing MDR-TB treatment over the last 12-month period					
**HIV**	**Both sexes**	**M**	**F**	**Pregnant**	
Number of unique individuals with an active HIV diagnosis (ICD code B20)					
Number of unique individuals with HIV who received treatment over the last 12-month period					
Number of unique individuals completing HIV treatment over the last 12-month period					
**Hepatitis C**	**Both sexes**	**M**	**F**	**Pregnant**	
Number of unique individuals with chronic HCV infection (HCV RNA positive) (ICD code B18.2)					
Number of unique individuals with chronic HCV infection (HCV RNA) who received antiviral treatment over the last 12-month period					
Number of unique individuals who following antiviral treatment achieved sustained viral response over the last 12-month period					
**Hepatitis B**	**Both**	**M**	**F**	**Pregnant**	

	sexes				
Number of unique individuals with chronic HBV (HBsAg) (ICD code B18.0–18.1)					
Number of unique individuals with chronic HBV infection (HBsAg) who are receiving care whilst in prison (treatment or long term follow up)					
Number of unique individuals with chronic HBV infection (HBsAg) and eligible for treatment (according to international treatment guidelines) who received antiviral treatment over the last 12-month period					
**Sexually Transmitted Infections (STIs) not formerly mentioned (incl. gonorrhoea, chlamydia, syphilis, and genital herpes)**	**Both sexes**	**M**	**F**	**Pregnant**	
Number of unique individuals with an STI diagnosis over the last 12-month period					
Number of unique individuals with STIs who received treatment over the last 12-month period					
Number of unique individuals completing STI treatment over the last 12-month period					
**COVID-19**	**Both sexes**	**M**	**F**		
Number of unique individuals with a SARS-Co-V2 infection laboratory confirmed					
**Oral health**	**Both sexes**	**M**	**F**		
Number unique individuals with oral health visit over the last 12-month period					
Number of unique individuals keeping 21 or more natural teeth					
**Mental health disorders**	**Both sexes**	**M**	**F**		
Number of unique individuals with a mental disorder diagnosis (ICD code F01-F99) on record. Diagnosis made either prior to incarceration or while in prison					
Number of unique individuals with a psychotic disorder diagnosis (ICD code F20-29) on record. Diagnosis made either prior to incarceration or while in prison.					
Number of unique individuals with recorded suicide attempt events (ICD code T14-91) in the last 12-month period					
Number of unique individuals who have received treatment for any mental health disorder over the last 12-month period					
**Substance Use Disorders**	**Both sexes**	**M**	**F**		
Number of unique individuals considered to have an active drug use disorder in the last 12-month period					
Number of unique individuals who have received pharmacological treatment for a substance use disorder over the last 12-month period					
Number of unique individuals who have received pharmacological treatment for an opioid use					

disorder over the last 12-month period					
**Diabetes Mellitus**	**Both sexes**	**M**	**F**		
Number of unique individuals with a diabetes mellitus diagnosis (ICD code E08-E13) on record. Diagnosis made either prior to incarceration or while in prison.					
Number of unique individuals with a diabetes mellitus diagnosis (ICD code E08-E13) who had at least two routine healthcare visits (excluding ophthalmology and other specialty visits) over the last 12-month period					
Number of unique individuals with a diabetes mellitus diagnosis (ICD code E08-E13) who had at least one ophthalmology visit over the last 12-month period					
Number of unique individuals who have received pharmacological treatment for diabetes over the last 12-month period					
**Hypertension**	**Both sexes**	**M**	**F**		
Number of unique individuals with a hypertension diagnosis (ICD code I10-I16) on record. Diagnosis made either prior to incarceration or while in prison.					
Number of unique individuals who have received pharmacological treatment for hypertension over the last 12-month period					
**Cardiovascular Disease**	**Both sexes**	**M**	**F**		
Number of unique individuals with a diagnosis for cardiovascular disease (CVD) on record. Diagnosis made either prior to incarceration or while in prison. This includes Cardiovascular and Ischemic Disease – ICD code I20-I25, I26-I28, I30-I52 AND Circulatory System Diseases – ICD code I60-I79)					
Number of unique individuals with a CVD diagnosis who had at least one routine healthcare visit over the last 12-month period					
Number of unique individuals who have received pharmacological treatment for cardiovascular disease over the last 12-month period					
**Cancer**	**Both sexes**	**M**	**F**		
Number unique individuals with a cancer diagnosis (ICD codes C00-D48) on record. Diagnosis made either prior to incarceration or while in prison.					
Number of unique individuals who have received treatment for cancer over the last 12-month period					

SECTION E: PRISON ENVIRONMENT

E1. Please indicate the number of individuals put in solitary confinement for behaviour or security reasons (one or more times during the last 12-month period):

E2. Do all people in prison have access to a toilet in-cell?

a) In all prisons
b) In most prisons
c) In a minority of prisons
d) In no prisons

E3. Do all people have access to shower & bathing facilities, with water at a temperature suitable to the climate?

a) In all prisons
b) In most prisons
c) In a minority of prisons
d) In no prisons

E4. Are there any facilities available for physical activity (e.g., gym, indoors or outdoors)?

a) In all prisons
b) In most prisons
c) In a minority of prisons
d) In no prisons

E5. Are people given the opportunity to use these facilities at least once a week?

a) In all prisons
b) In most prisons
c) In a minority of prisons
d) In no prisons

E6. Are people in prison given the chance to spend at least one hour per day outdoors?

a) In all prisons
b) In most prisons
c) In a minority of prisons
d) In no prisons

E7. Are diets in prison adapted to cultural needs, by giving at least two options of food?

a) In all prisons
b) In most prisons
c) In a minority of prisons
d) In no prisons

E8. Are diets adapted to gender needs (i.e., number of calories varies between female and male prisons)?

a) Yes
b) No

SECTION F: HEALTH BEHAVIOURS

F1. Please provide the following numbers in reference to the situation observed by 31.12.2020.

	Both sexes	Male	Female	No data
Number of unique individuals with BMI>=25				☐
Number of unique individuals with BMI>=30				☐
Number of unique individuals who currently use tobacco products				☐
Number of unique individuals who drink/have drank alcohol (last 12 months)				☐
Number unique individuals who use/have used drugs (last 12 months)				☐
Number of unique individuals who inject/have injected drugs (over the last 12 months)				☐
Number of unique individuals who regularly exercise for a minimum of 150 minutes/week				☐

SECTION G: ADHERENCE TO THE PRINCIPLE OF EQUIVALENCE AND OTHER INTERNATIONAL STANDARDS

G1. Do health care services for people in prisons offer the same scope of services as in the community?
a) Yes
b) No

G2. Is there a national vaccine implementation plan establishing the access for people in prison to COVID-19 vaccine?
a) Yes, people in prison are considered one of the priority groups.
b) Yes, the principle of equivalence is followed, so the same priority groups identified in the general community are prioritised in prison (i.e., elderly and healthcare staff, by phases)
c) Yes, people in prison are referred to in this plan but are only considered after all people in the community are immunized
d) Unsure, people in prison are not mentioned in the national vaccine implementation plan
e) We have no national vaccine implementation plan established

G2.1 If the answer to G2 is a, please indicate the level of priority attributed (open question):

G3. Are health-care services in prisons subject to the same standards and accreditation procedures as health care services in the community?
a) Yes, for publicly contracted services only
b) Yes, for both public and private services
c) No

G4. Is the prison health workforce subject to the same professional standard as the health workforce in the community?
a) Yes
b) No

G5. Is the prison health workforce subject to the same ethical standard as the health workforce in the community?
a) Yes
b) No

G6. Are the provisions of international law regarding the health of people in prisons and other places of detention incorporated into national law?
a) Yes
b) No

G7. Can clinical decisions taken by health staff be overruled or ignored by non-health prison staff?
a) Yes
b) No

G8. Are there publicly available reports of prison hygiene, nutrition and living conditions?

a) Yes. If yes, please provide a link to the latest one:
b) No

G9. Does a national healthcare complaints system exist in your country, and is it available to prisoners?

a) Yes
b) No

G9.1 If the answer to G9 is ‘Yes’, please indicate how many complaints were received in the last 12-month period and provide a link to the latest one.

Number of complaints	
Weblink	

SECTION H: REDUCING HEALTH INEQUALITIES AND ADDRESSING THE NEEDS OF SPECIAL POPULATIONS

H1. Are there national standards to meet the health needs of special populations in prison? (select all that apply)

- o Women
- o Pregnant
- o Children and youth
- o LGBTIQ persons
- o Foreign nationals
- o People who use drugs
- o Elderly prisoners
- o People with physical disabilities
- o People with learning disabilities
- o Ethnic minorities
- o None of the above (*exclusive choice*)

H2. Are any of the national standards to meet the health needs of special populations based on relevant international standards?

a) Yes
b) No

H3. Do prisons have health related information products for people in prison such as brochures and leaflets in multiple languages?

a) In all prisons
b) In most prisons
c) In a minority of prisons
d) In no prisons

H4. Do women in prison have the option to be attended by female health care staff?

a) Yes
b) No

H5. Are women offered a pregnancy test on admission to prison?

a) Yes, only once
b) Yes, and they are repeated at regular intervals
c) No

H5.1. If the answer to H5 is a) or b), are women offered the possibility of prenatal care or termination, in case of a positive result?

a) Yes
b) No

H6. How many women gave birth whilst in prison in the last 12 months?

Thank you very much for your assistance!

Please give any other comments to this survey:

